# Use of low-molecular-weight heparin in severe paraquat poisoning: a case report

**DOI:** 10.1186/s13256-020-02565-9

**Published:** 2020-12-08

**Authors:** Maria A. Montoya-Giraldo, Luisa F. Díaz, Ubier E. Gómez, Juliana Quintero, Andres F. Zuluaga

**Affiliations:** 1 CIEMTO [Drug and Poison Research and Information Center], Calle 64 # 51-31, Laboratorio Integrado de Medicina Especializada (LIME), IPS Universitaria, Facultad de Medicina, Universidad de Antioquia, Medellín, Antioquia Colombia; 2 Hospital Universitario San Vicente Fundación, Medellín, Colombia

**Keywords:** Paraquat, Toxicity, Poisoning, Enoxaparin

## Abstract

**Background:**

Acute paraquat ingestion remains a leading cause of mortality in developing countries. There is currently no evidence that treatment with high-dose immunosuppressants and antioxidants improves survival in patients with paraquat poisoning, and better options are urgently needed. Here, we describe the unexpected survival and recovery of a patient with a potentially fatal paraquat poisoning.

**Case presentation:**

After ingesting 28 mL of paraquat (20% ion w/v), confirmed by a deep blue color in the urine dithionite test (UDT), a 17-year-old Hispanic Colombian boy was treated according to the hospital protocol with cyclophosphamide, methylprednisolone, *N*-acetylcysteine, vitamin E and propranolol. Gastrointestinal endoscopy showed extensive ulceration and necrosis. As a novelty, enoxaparin at a single dose of 60 mg was added to his treatment. Despite the evidence of severe mucosal burns in the gastrointestinal tract and high paraquat concentrations found in the UDT, the clinical condition began to improve after 1 day of treatment, with full recovery and discharge from hospital after 21 days.

**Conclusions:**

Although the amount of paraquat ingested by the patient was large and the UDT indicated severe poisoning with a somber prognosis, unexpected survival of the patient was observed, and the addition of enoxaparin was the only change from the standard treatment.

## Introduction

Paraquat (PQ) self-ingestion remains a leading cause of pesticide-induced mortality [1]. In Colombia, the burden of PQ intoxication between 2010 and 2016 was higher than that reported for all other chemicals, reaching 53.4 disability-adjusted life years (DALY) per 100,000 inhabitants [2].

Besides the accurate characterization of the patient’s exposure, quantification of the plasma PQ concentration has remarkable value for assessing the prognosis [3]. Although liquid chromatographic methods are the gold standard, their availability in emergency rooms is limited [4]. For this reason, a reliable and suitable rapid test such as the semiquantitative urine dithionite test (UDT) is preferred [3]. While the cutoff value for UDT is 2 mg/L, a deep blue color correlates with plasma concentrations > 10 mg/L and 100% early lethality [3–5]. Patients usually receive high-dose immunosuppressants combined with antioxidants, but there is no evidence that this treatment improves survival in severe PQ poisoning [6, 7], and better therapeutic options are urgently needed. Thrombotic microangiopathy was recently elucidated as the primary systemic pathological event in PQ poisoning [8], but antithrombotic drugs have never been tested. Here, we describe the unexpected survival of a patient with severe PQ poisoning after adding enoxaparin to his treatment.

## Case presentation

A 17-year-old Hispanic Colombian boy from a rural area, troubled by familial and financial issues, tried to kill himself by allegedly consuming 28 mL of PQ (Gramoxone SL 1 L, 200 g/L ion PQ), equivalent to a high dose of approximately 80 mg/kg of PQ ion [9]. Shortly after the ingestion he experienced a repulsive taste, severe odynophagia, dysphagia, stomach pain and vomiting. He was given kitchen oil and *panela* by his mother. Two hours had already passed when the patient arrived at the emergency department of a local hospital. Gastric lavage was done with activated charcoal. The patient also received vitamin E and *N*-acetyl cysteine (NAC). He was then referred to the Hospital San Vicente Foundation (Medellin, Colombia) for further management.

**Fig. 1:**
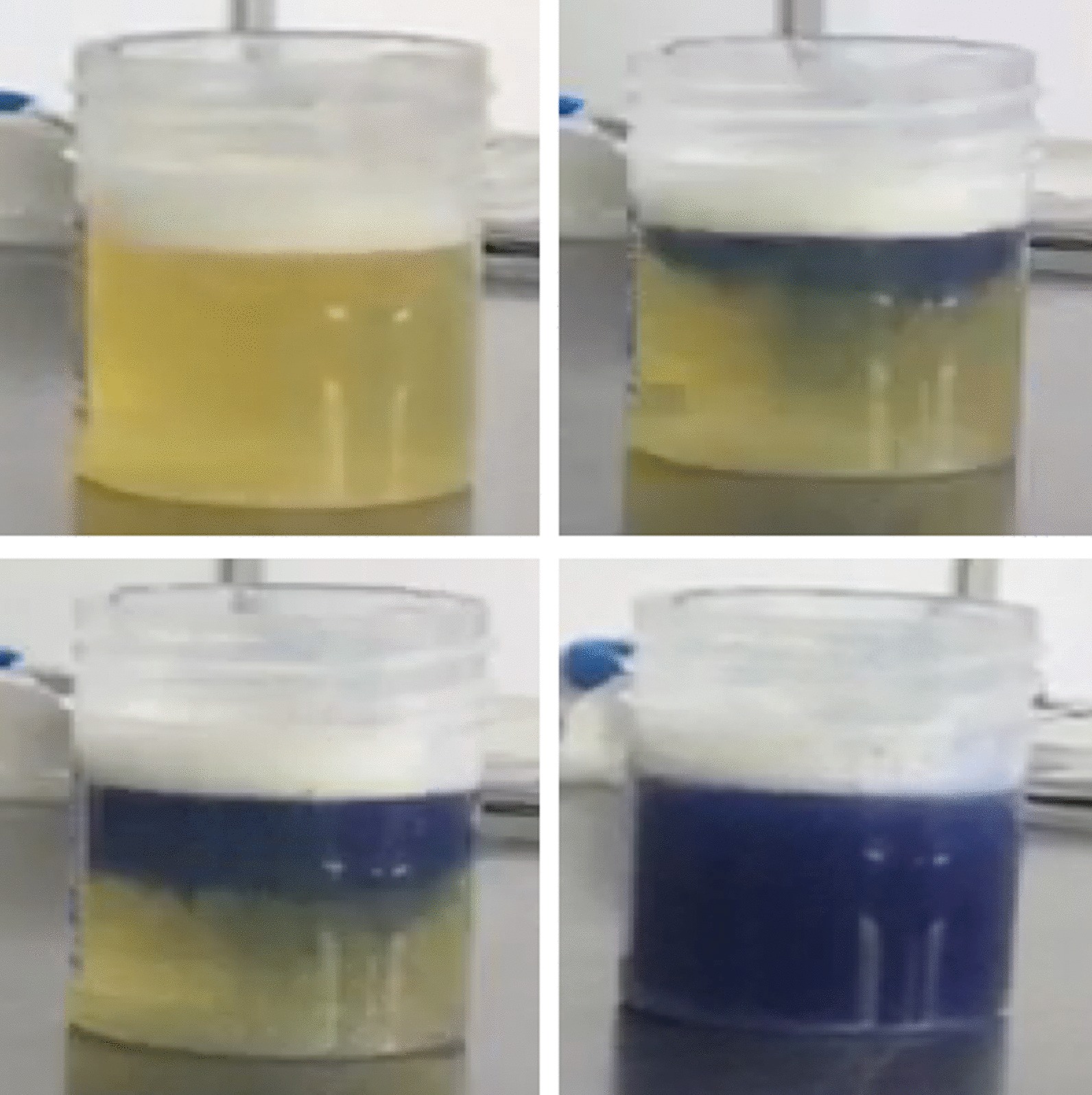
The qualitative dithionite test in urine revealed a deep blue color

**Fig. 2:**
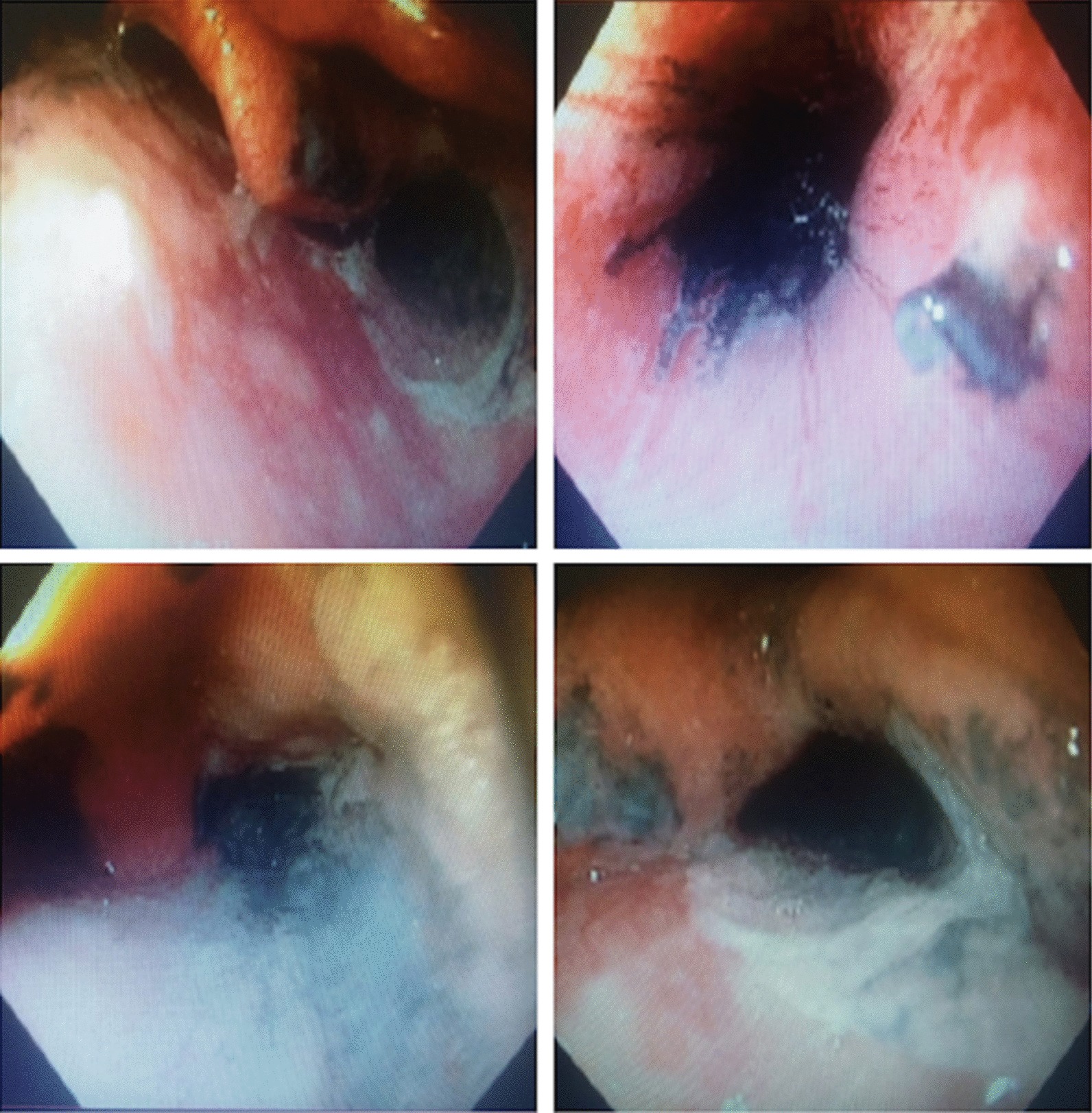
Endoscopy of upper digestive tract. Supraglottic cricopharyngeal and mucosal structures of the middle third of the esophagus, with extensive and multiple ulcers covered with fibrin and hematin, with some areas of necrotic appearance

On arrival 9 hours post-ingestion, the patient had arterial pressure of 123/65 mmHg, pulse rate of 78 per minute, respiratory rate of 16 per minute and 100% oxygen saturation. The following treatment was ordered: NAC 1.8 g in 500 mL of 5% dextrose to infuse over 1 hour, vitamin E 400 mg orally every 12 hours, propranolol 40 mg orally every 8 hours, methylprednisolone 1 g intravenously every 24 hours, cyclophosphamide 900 mg in 500 mL of 5% dextrose to be infused over 1 hour, and omeprazole 40 mg intravenously every 12 hours. Laboratory tests showed a white blood cell count of 11,750/mm^3^ (80% neutrophils), hemoglobin 16.6 g/dL and hematocrit 50%, platelets 428,000/mm^3^, prothrombin time 7 seconds, and activated partial thromboplastin time of 42 seconds. Electrocardiogram, liver enzyme and kidney function tests were normal. UDT showed a dark blue color at 12 hours post-ingestion (Fig. 1). Considering the large amount of PQ ingested, the positive UDT and the unavailability of hemoperfusion, the family was informed of the patient’s poor prognosis. Based on reports of thrombotic microangiopathy as the primary systemic pathological event in PQ poisoning, a single dose of enoxaparin (60 mg subcutaneously) as an extraordinary measure was offered, to which the patient consented. As severe gastroesophageal chemical burns were found by endoscopy 84 hours post-ingestion (Fig. 2), the oral route was stopped for a week. There were no signs or symptoms of pulmonary compromise during the hospital stay, but thoracic high-resolution computerized tomography showed minimal and unspecific atelectatic changes. A second endoscopy at day 14 after PQ ingestion was normal, showing no residual stenosis, and the patient was discharged in good condition at day 21. The patient was doing well at his 1-month health follow-up appointment.

## Discussion

Here we report the unexpected survival and full recovery of a patient with severe PQ poisoning after adding enoxaparin to the standard treatment.

Some factors may have had a positive role in the surprising survival of the patient: the unknown true amount of PQ ingested, receiving basic detoxification measures within a couple of hours, young age and good baseline health. However, PQ intoxication commonly affects healthy Colombian men ranging in age from 15 to 44 years [2], and it is well accepted that the ingestion of > 40 mg PQ ion/kg of body weight results in early death (24–48 hours) from multiple organ failure, independently of the treatment used [6, 7]. Moreover, with a highly positive UDT (dark blue color) after 12 hours or more, the extensive gastrointestinal damage observed by endoscopy suggests that the absorption of PQ was almost complete, and detoxification measures were insufficient to explain the weakness of clinical presentation. Similar cases are frequently treated in our institution [10], but the prognosis in almost all cases correlates well with UDT level. Since 1979, it has been accepted that a plasma PQ concentration higher than 1 mg/L at 12 hours after poisoning correlates with mortality of at least 80% [11, 12]. In 1987, Scherrmann et al. evaluated the correlation between urine and plasma PQ concentrations [5], demonstrating that a dark blue color in UDT correlates with fatal plasma concentrations higher than 10 mg/L [13]. The lack of a confirmatory method such as gas chromatography was probably a limitation of our study, but it is accepted that a false-positive result in the urine dithionite test is unlikely, although there are cross-reactions with bilirubin at high levels [3]. In fact, it is possible to use this colorimetric method to distinguish between bipyridyl compounds: diquat (green color) and PQ (blue color) [14].

It is commonly accepted that PQ exerts injurious effects through oxidative stress and mitochondrial dysfunction [15], but as therapy with antioxidants and immunosuppressants has failed in clinical trials [6], other therapeutic options are needed. Although enoxaparin cannot be deemed directly responsible for the favorable evolution, recent experimental data support its biological plausibility, which deserves to be discussed in depth.

Pulmonary capillary endothelial cells are the direct targets of PQ toxicity after ingestion [16–18]. Recent studies have shown the role of the platelet endothelial cell adhesion molecule-1 (PECAM-1 or cluster of differentiation 31 [CD31]) in PQ poisoning. PECAM-1, a key member of the immunoglobulin superfamily adhesion protein, is a specific regulator of the endothelial junctional integrity [19] regulating vascular permeability and leukocyte exudation. Interestingly, PECAM-1 was found to be markedly reduced in PQ poisoning during acute lung inflammation in rabbits [20]. Heparin and related drugs have been shown to bind the extracellular domain of PECAM-1 and also heparan sulfate on cell surfaces under conditions of mild acidosis [21, 22]. If the amelioration of the endothelial injury is due to the enoxaparin binding with PECAM-1, hampering its binding to PQ is a hypothesis that deserves to be tested in the future.

It is increasingly recognized that low-molecular-weight heparins (LMWH) have many pharmacological properties beyond their anticoagulant activity [21] [23]. As a naturally occurring molecule, endogenous heparin is specifically localized to immune mast cells in humans to physiologically modulate certain inflammatory pathways [24], for example through inhibition of the complement cascade. The complement system is a pivotal component of the innate immune system in the response against microorganisms, and it is dysregulated by toxic agents such as PQ [25]. Complement can be activated by three distinct pathways: the classical, alternative and lectin pathways. The specific heparin–complement protein interactions include inhibition of C1q (initiator of the classical complement pathway), augmentation of factor H (soluble inhibitor of the alternative pathway) and inhibition of the lytic terminal complement complex [24]. The three pathways converge at the cleavage and activation of C3, generating split products with biological effector functions (C3 opsonins, cytolytic membrane attack complex, and so on). In 2011, Sun demonstrated the key role of the complement factor H (CFH) in paraquat-induced acute lung injury in PQ poisoning in mice [25, 26]. As LMWH have the strongest polyanionic negative charge known, binding to CFH is expected [23, 27, 28]. If the LMWH-CFH complex is a damage-limiting mechanism, downregulation of the alternative pathway in PQ poisoning is another hypothesis that deserves further testing [29, 30].

Remarkably, endothelial cell injury and thrombus formation in the microvascular environment in humans is called thrombotic microangiopathy (TMA), and drug-induced TMA is complement-mediated [31]. Daisley et al. recently reported the histological examination of lungs from the autopsy of a PQ-poisoned patient [8]. The main finding was that pulmonary thrombotic microangiopathy was the primary pathological event in PQ poisoning, characterized by alveolar capillary thrombosis, atelectatic changes, destruction of alveolar walls, rupture of subpleural alveolar walls and emphysematous changes [8]. Therefore, the potential for antithrombotic drugs in severe cases deserves to be tested [32].

Here, we chose enoxaparin for specific reasons. First, enoxaparin has more predictable pharmacokinetics than unfractionated heparin (UFH), and it has been used extensively in intensive care units, requiring a subcutaneous daily dose of 60 mg [16]. Second, the hemopurification strategy for acute PQ poisoning in adults involves the use of enoxaparin [17]. Third, this drug has been tested in an animal model to prevent lung fibrosis induced by PQ [18].

Finally, regarding the dose, a daily dose of 40 mg of enoxaparin has been used in pregnant women with a history of thromboembolic events during previous pregnancies, reducing the complement activity *in vivo* [19], but other anticoagulants (such as fondaparinux and hirudin) do not inhibit the generation of complement split products [20]. Moreover, Robinson et al. compared the effective dosage of enoxaparin for intensive care patients in 72 patients randomized in four groups to receive 40, 50, 60 or 70 mg subcutaneously for a period of 24 hours, measuring the anti-factor Xa (aFXa) as evidence of antithrombotic activity, which demonstrated a ceiling effect at 60 mg in a single dose [21].

## Conclusion

Although the ingested amount of paraquat by the patient was large and the UDT indicated severe poisoning with a somber prognosis, unexpected survival of the patient was observed, and the addition of enoxaparin was the only change from the standard treatment. As there are many methodological flaws and confounders in this case report, more and better data are required to corroborate our observation.

## Data Availability

All data generated or analyzed during this study are included in this article.
